# Citrate synthase from *Synechocystis* is a distinct class of bacterial citrate synthase

**DOI:** 10.1038/s41598-019-42659-z

**Published:** 2019-04-15

**Authors:** Shoki Ito, Naoto Koyama, Takashi Osanai

**Affiliations:** School of Agriculture, Meiji University, 1-1-1, Higashimita, Tama-ku, Kawasaki, Kanagawa 214-8571 Japan

## Abstract

Citrate synthase (CS, EC 2.3.3.1) catalyses the initial reaction of the tricarboxylic acid (TCA) cycle. Although CSs from heterotrophic bacteria have been extensively studied, cyanobacterial CSs are not well-understood. Cyanobacteria can produce various metabolites from carbon dioxide. *Synechocystis* sp. PCC 6803 (*Synechocystis* 6803) is a cyanobacterium used to synthesize metabolites through metabolic engineering techniques. The production of acetyl-CoA-derived metabolites in *Synechocystis* 6803 has been widely examined. However, the biochemical mechanisms of reactions involving acetyl-CoA in *Synechocystis* 6803 are poorly understood. We characterised the CS from *Synechocystis* 6803 (*Sy*CS) and compared its characteristics with other bacterial CSs. *Sy*CS catalysed only the generation of citrate, and did not catalyse the cleavage of citrate. It is suggested that *Sy*CS is not related to the reductive TCA cycle. The substrate affinity and turnover number of *Sy*CS were lower than those of CSs from heterotrophic bacteria. *Sy*CS was activated by MgCl_2_ and CaCl_2_, which inhibit various bacterial CSs. *Sy*CS was not inhibited by ATP and NADH; which are typical feedback inhibitors of other bacterial CSs. *Sy*CS was inhibited by phosphoenolpyruvate and activated by ADP, which has not been reported for CSs from heterotrophic bacteria. Thus, *Sy*CS showed unique characteristics, particularly its sensitivity to effectors.

## Introduction

Citrate synthase (CS, EC 2.3.3.1) catalyses the first reaction of the tricarboxylic acid (TCA) cycle: oxaloacetate + acetyl-CoA + H_2_O → citrate + CoA-SH. Different bacterial CSs have been characterised, and studies have shown that the biochemical properties of CSs from Gram-positive bacteria and Gram-negative bacteria exhibit some differences. CSs from Gram-negative bacteria are inhibited by NADH, whereas CSs from Gram-positive bacteria are not^1,2^. However, the biochemical properties of cyanobacterial CSs have not been well-characterised.

**Figure 1:**
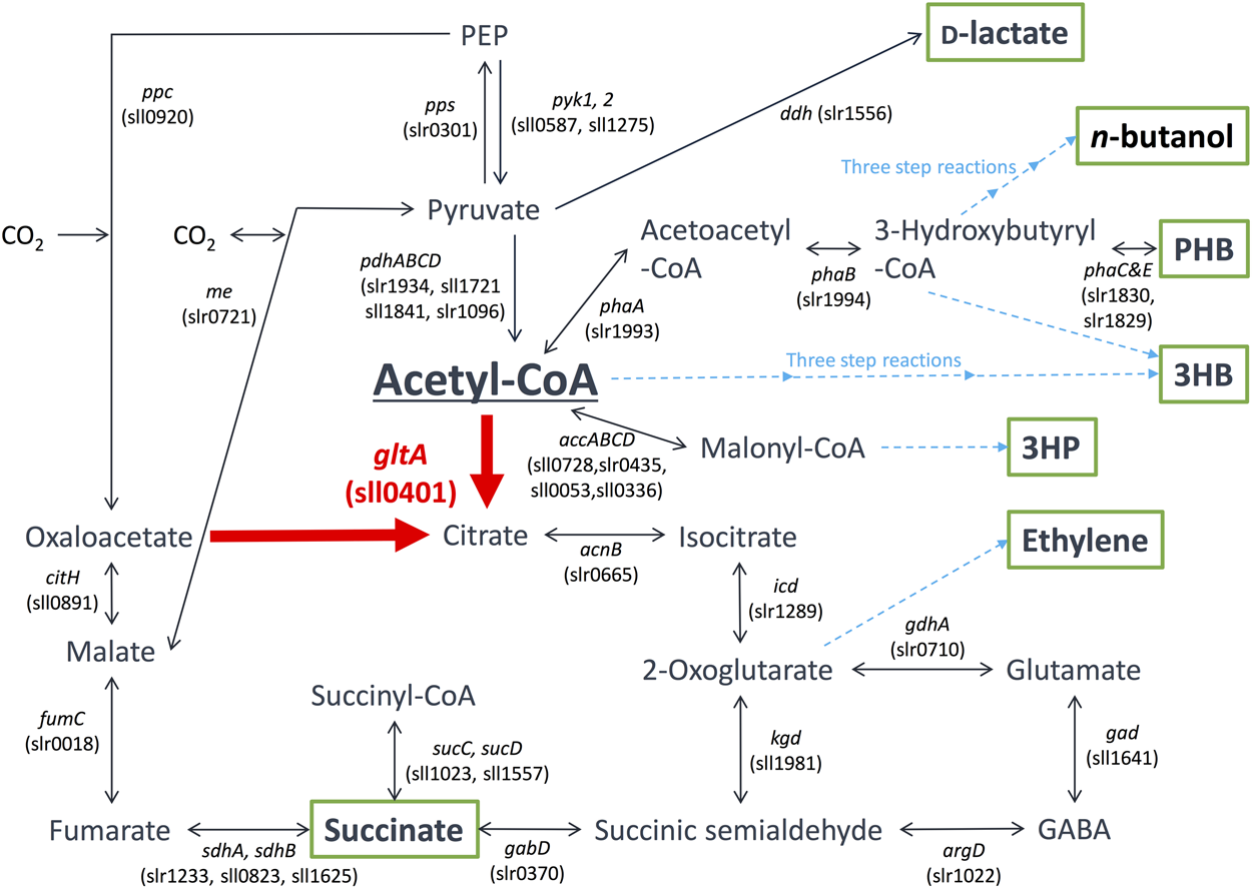
Metabolic map around the TCA cycle in *Synechocystis* sp. PCC 6803. The information of genes encoding enzymes in *Synechocystis* sp. PCC 6803 were obtained by Kyoto Encyclopedia of Genes and Genomes (KEGG) database (http://www.genome.jp/kegg/). The green squares represent the useful metabolites having been reported previously. The blue dotted lines represent the synthetic reactions being catalysed by foreign enzymes. 3HB: 3-hydroxybutyrate, 3HP: 3-hydroxypropionic acid, PHB: polyhydroxybutyrate.

Photoautotrophic cyanobacteria have been evaluated for use in the sustainable production of bioplastics and biofuels from carbon dioxide using light energy^3^. *Synechocystis* sp. PCC 6803 (hereafter, *Synechocystis* 6803) is a unicellular cyanobacterium that has been widely studied for use in metabolite production. *Synechocystis* 6803 exhibits suitable characteristics as a host for metabolite production such as rapid growth and natural transformation capability^4^. *Synechocystis* 6803 can be repeatedly genetically manipulated^5^. Various genetic toolsets have been developed for *Synechocystis* 6803^6^. This freshwater cyanobacterium can also grow in artificial seawater supplemented with nitrogen and phosphorus^7^. Under dark and anaerobic conditions, *Synechocystis* 6803 produces organic acids such as d-lactate and succinate, which are used in bioplastics production (Fig. 1)^8^. In *Synechocystis* 6803, biochemical analyses of a phosphoenolpyruvate carboxylase (encoded by *pps*, sll0920) being the rate-limiting enzyme in succinate production^9,10^ and d-lactate dehydrogenase (encoded by *ddh*, slr1556) catalysing the final reaction in d-lactate production have been performed (Fig. 1)^11,12^. These reports identified the enzymatic properties and the critical amino acid residues regulating the enzymatic activities, and emphasised the importance of biochemical studies of this cyanobacterium^11,12^. For example, we have previously indicated that lower activity of d-lactate dehydrogenase (Ddh) from *Synechocystis* 6803 compared to other bacterial Ddh is one of the weak point of d-lactate production using *Synechocystis* 6803^12^. We have indicated that Ddh from *Synechocystis* 6803 also catalyses the reaction from oxaloacetate to malate and an amino acid substitution alters the substrate specificity of Ddh^12^.

Characterising acetyl-CoA metabolism and the enzymes involved is crucial for understanding the basic science of cyanobacteria and its potential practical applications. The intracellular acetyl-CoA concentration of *Synechocystis* 6803 is much higher compared to those of other cyanobacteria^13^. The production of polyhydroxybutyrate (PHB)^14–16^, 3-hydroxybutyrate (3HB)^17^, *n*-butanol^18^, and 3-hydroxypropionic acid (3HP)^19,20^ from acetyl-CoA in *Synechocystis* 6803 has been studied (Fig. 1). Ethylene production from the TCA cycle intermediates in *Synechocystis* 6803 has also been demonstrated (Fig. 1)^21,22^. Despite these metabolic engineering applications, few studies have conducted biochemical analysis using acetyl-CoA as a substrate in cyanobacterium. In this study, we performed biochemical analyses of *Sy*CS (encoded by *gltA*, sll0401) (Fig. 1) and compared its properties with those of other bacterial CSs.

## Results

### Purification and kinetic analyses of *Sy*CS

**Figure 2:**
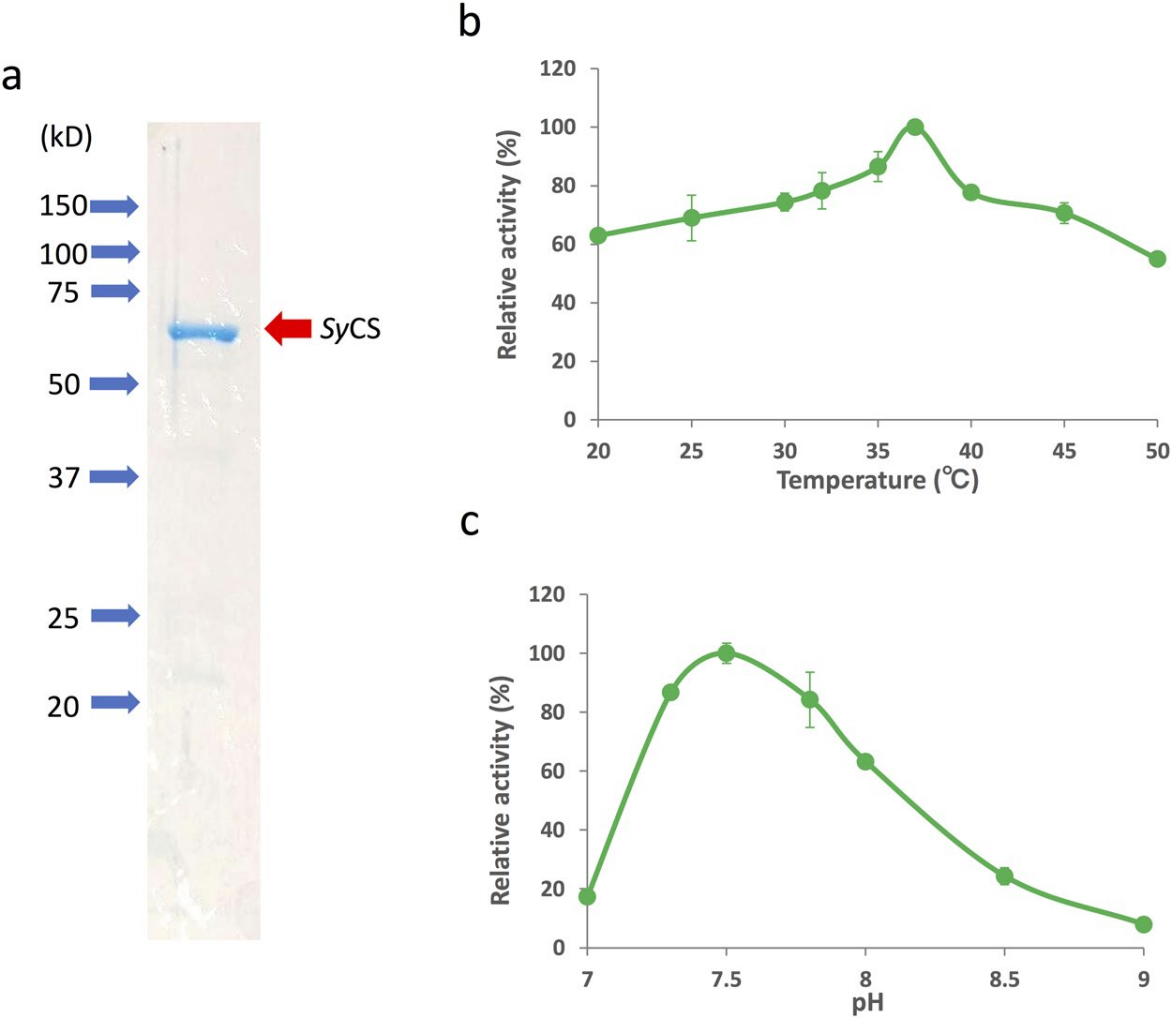
Effect of temperature and pH on *Sy*CS activity. (**a**) SDS-PAGE analysis after affinity purification of GST-tagged *Sy*CS. The purified proteins were electrophoresed by 12% SDS-PAGE and stained with InstantBlue reagent. Arrows (blue) indicate the positions of molecular protein markers. (**b**) *Sy*CS activity at different temperatures. The experiment was conducted using 150 pmol of *Sy*CS at pH 7.5. The concentrations of oxaloacetate and acetyl-CoA were 1 and 0.5 mM, respectively. *Sy*CS activity was set to 100% at 37 °C. The data represent the mean ± SD from three independent experiments. (**c**) *Sy*CS activity at different pH values. The experiment was conducted using 150 pmol of *Sy*CS, and the temperature was 37 °C. The concentrations of oxaloacetate and acetyl-CoA were 1 and 0.5 mM, respectively. *Sy*CS activity was set to 100% at pH 7.5. The data represent the mean ± SD from three independent experiments.

**Figure 3:**
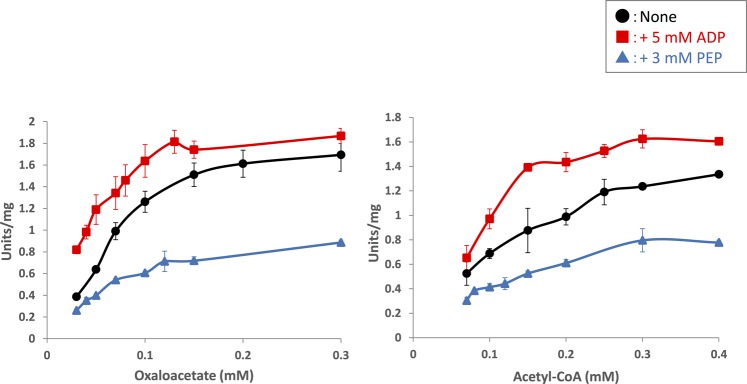
Saturation curves of *Sy*CS for oxaloacetate (left) and acetyl-CoA (right). The experiment was conducted using 50 pmol of *Sy*CS at 37 °C, pH 7.5. The concentrations of oxaloacetate and acetyl-CoA were set to 1 and 0.5 mM, respectively. The data represent the mean ± SD from three independent experiments.

For biochemical analysis of *Sy*CS, glutathione-*S*-transferase (GST)-tagged *Sy*CS was purified from *Escherichia coli* by affinity chromatography (Fig. 2a). The optimal conditions at which *Sy*CS showed maximal activity were 37 °C and pH 7.5 (Fig. 2b,c). Therefore, subsequent analysis of *Sy*CS was conducted under these conditions. To calculate the kinetic parameters of *Sy*CS, saturation curves of *Sy*CS for both substrates were drawn (Fig. 3). The observed *K*_m_ value of *Sy*CS for oxaloacetate was 91 ± 11 μM and the *k*_cat_ value for oxaloacetate was 2.76 ± 0.26 s^−1^ (Table 1). The *k*_cat_/*K*_m_ value of *Sy*CS for oxaloacetate was 30.50 ± 3.17 s^−1^mM^−1^ (Table 1). Similarly, for acetyl-CoA, the *K*_m_ value was 220 ± 77 μM and the *k*_cat_ value was 2.51 ± 0.24 s^−1^ (Table 1). The *k*_cat_/*K*_m_ value of *Sy*CS for acetyl-CoA was 12.07 ± 2.95 s^−1^mM^−1^ (Table 1). *Sy*CS showed no catalytic activity for the backward reaction, generating acetyl-CoA and oxaloacetate from citrate and CoA.

**Table 1:** Kinetic parameters of *Sy*CS for oxaloacetate and acetyl-CoA.

Substrate	Effector	*K*_m_ (μM)	*k*_cat_ (s^−1^)	*k*_cat_/*K*_m_ (s^−1^mM^−1^)
Oxaloacetate	None	91 ± 11	2.76 ± 0.26	30.50 ± 3.17
	5 mM ADP	73 ± 11	3.35 ± 0.54	45.65 ± 0.84^*^
	3 mM PEP	108 ± 4	1.53 ± 0.06^*^	14.19 ± 0.99^*^
Acetyl-CoA	None	220 ± 77	2.51 ± 0.24	12.07 ± 2.95
	5 mM ADP	153 ± 24	2.99 ± 0.12	19.90 ± 2.82^*^
	3 mM PEP	194 ± 36	1.42 ± 0.18^*^	7.39 ± 0.70

The experiment was conducted using 50 pmol of *Sy*CS, and enzymatic activity was measured under optimal conditions (37 °C and pH 7.5). The concentrations of oxaloacetate or acetyl-CoA were 1 and 0.5 mM, respectively. The data represent the mean ± SD from three independent experiments. The asterisks indicated significant differences between the absence and presence of the effector (Student’s *t* test; *P < 0.05).

### Effects of ions on *Sy*CS activity

**Figure 4:**
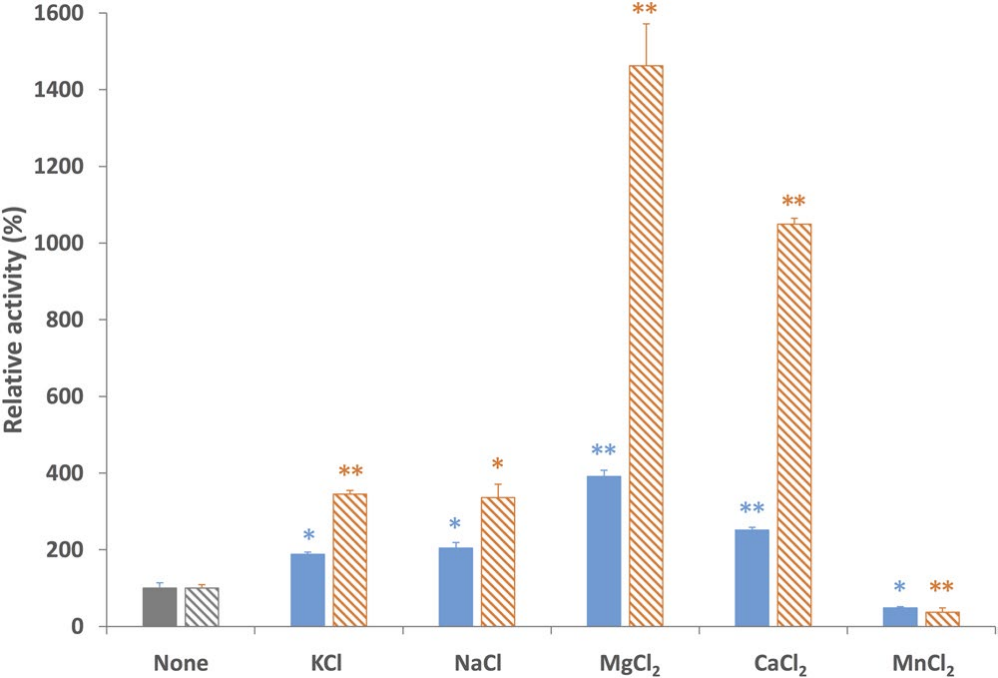
Effect of monovalent and divalent salts on *Sy*CS activity. The experiment was conducted using 50 pmol of *Sy*CS at 37 °C, pH 7.5. The concentrations of monovalent and divalent salts were set to 100 mM. Oxaloacetate and acetyl-CoA were set to the saturated concentrations (left bars; 1 and 0.5 mM, respectively), and the *K*_m_ values (right bars; 0.091 and 0.220 mM, respectively). *Sy*CS activity in the absence of monovalent and divalent salts was set to 100%. The data represent the mean ± SD from three independent experiments. The asterisks indicate significant differences between the absence and presence of the salt (Student’s *t* test; *P < 0.05, **P < 0.005).

The effects of monovalent and divalent salts on *Sy*CS activity were examined. *Sy*CS activity was altered by monovalent and divalent salts, which was affected by the concentration of substrates (Fig. 4). At a saturated concentration of both substrates in the absence of effectors, *Sy*CS activity increased to 188% in the presence of 100 mM KCl, 205% in the presence of 100 mM NaCl, 391% in the presence of 100 mM MgCl_2_, and 251% in the presence of 100 mM CaCl_2_, while enzyme activity was decreased to 48% in the presence of 100 mM MnCl_2_ (Fig. 4). At the half-saturated concentration of both substrates in the absence of effectors, *Sy*CS activity increased to 345% in the presence of 100 mM KCl, 336% in the presence of 100 mM NaCl, 1,463% in the presence of 100 mM MgCl_2_, and 1,050% in the presence of 100 mM CaCl_2_, while enzyme activity was decreased to 37% in the presence of 100 mM MnCl_2_ (Fig. 4).

**Figure 5:**
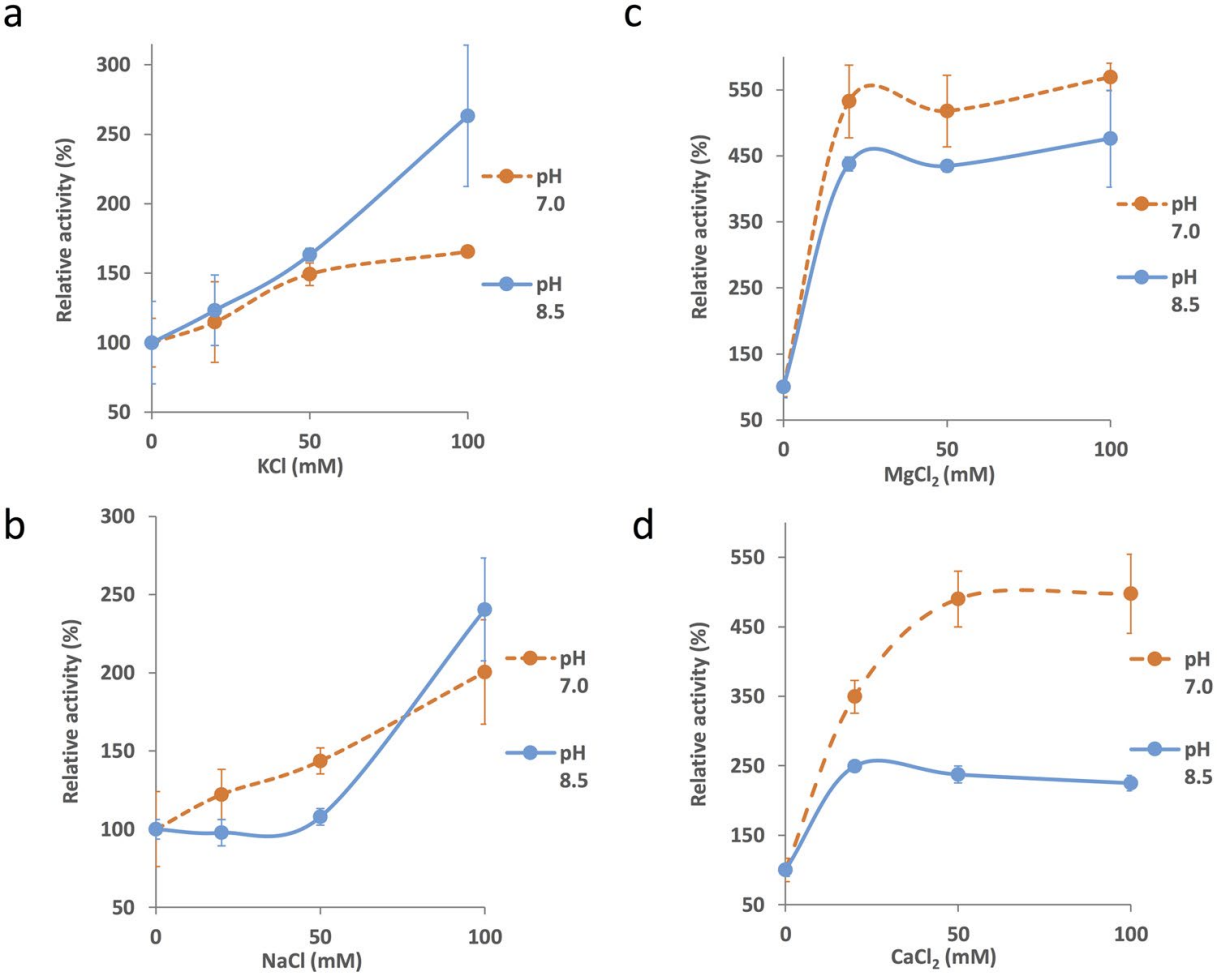
Effect of pH on activation of *Sy*CS by (**a**) KCl, (**b**) NaCl, (**c**) MgCl_2_, and (**d**) CaCl_2_. The experiment was conducted using 50 pmol of *Sy*CS at 37 °C, pH 7.0 or 8.5. The concentrations of oxaloacetate and acetyl-CoA were 1 and 0.5 mM. *Sy*CS activity in the absence of monovalent and divalent salts was set to 100%. The data represent the mean ± SD from three independent experiments.

The activation of *Sy*CS by monovalent and divalent salts was affected by the pH (Fig. 5). In KCl, *Sy*CS activation was concentration-dependent at both pH 7.0 and pH 8.5 (Fig. 5a). The activity of *Sy*CS increased linearly with increasing concentrations of NaCl at pH 7.0 (Fig. 5b). At pH 8.5, *Sy*CS activity did not increase up to 50 mM NaCl; however, it increased by 2.4-fold in the presence of 100 mM NaCl (Fig. 5b). The activation of *Sy*CS by 100 mM monovalent salts at pH 8.5 was greater than that at pH 7.0 (Fig. 5a,b). The activation of *Sy*CS peaked at 20 mM MgCl_2_ at both pH 7.0 and pH 8.5 (Fig. 5c). The activation of *Sy*CS peaked at 20 mM CaCl_2_ at pH 8.5 and at 50 mM CaCl_2_ at pH 7.0 (Fig. 5d). In contrast to monovalent salts, the activation of *Sy*CS by 100 mM divalent salts at pH 7.0 was greater than that at pH 8.5 (Fig. 5c,d).

### Regulation of *Sy*CS activity by metabolites

**Figure 6:**
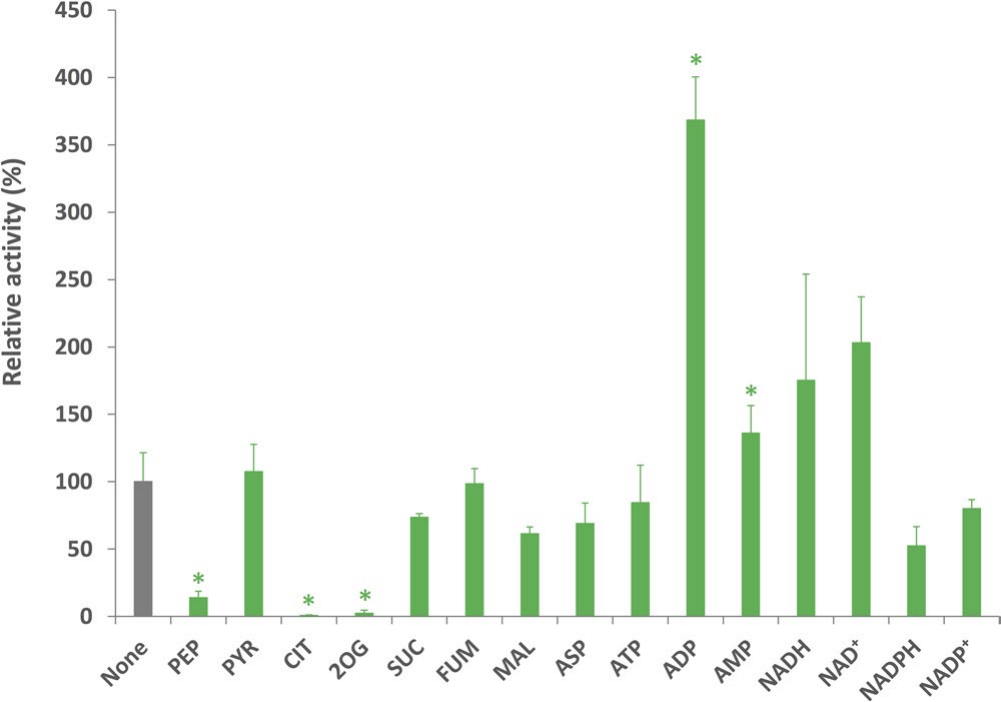
Effect of metabolites on *Sy*CS activity. The experiment was conducted using 50 pmol of *Sy*CS at 37 °C, pH 7.5. The concentrations of oxaloacetate and acetyl-CoA were the *K*_m_ values, 0.091 and 0.220 mM, respectively. Concentrations of the metabolites were 5 mM. *Sy*CS activity in the absence of metabolites was set to 100%. The data represent the mean ± SD from three independent experiments. PEP: Phosphoenolpyruvate, PYR: Pyruvate, CIT: Citrate, 2OG: 2-Oxoglutarate, SUC: Succinate, FUM: Fumarate, MAL: l-Malate, ASP: l-Aspartate. The asterisks indicate significant differences between the absence and presence of the metabolite (Student’s *t* test; *P < 0.05).

*Sy*CS activity was also altered by different metabolites (Fig. 6). In the presence of 5 mM phosphoenolpyruvate (PEP), citrate (CIT), and 2-oxoglutarate (2OG), *Sy*CS activity markedly decreased to 14%, 1%, and 2%, respectively (Fig. 6). Although PEP is a metabolite in glycolysis, it inhibited *Sy*CS activity (Fig. 6). To examine the changes in the kinetic parameters of *Sy*CS, saturation curves of *Sy*CS for both substrates in the presence of 3 mM PEP were drawn (Fig. 3). In the presence of 3 mM PEP, the *K*_m_ value of *Sy*CS for oxaloacetate was 108 ± 4 μM, the *k*_cat_ value of *Sy*CS for oxaloacetate decreased to 1.53 ± 0.06 s^−1^, and the *k*_cat_/*K*_m_ value of *Sy*CS for oxaloacetate decreased to 14.19 ± 0.99 s^−1^mM^−1^ (Table 1). In the presence of 3 mM PEP, the *K*_m_ value of *Sy*CS for acetyl-CoA was 194 ± 36 μM, the *k*_cat_ value of *Sy*CS for acetyl-CoA decreased to 1.42 ± 0.18 s^−1^, and the *k*_cat_/*K*_m_ value of *Sy*CS for acetyl-CoA was 7.39 ± 0.70 s^−1^mM^−1^ (Table 1). In contrast, *Sy*CS activity significantly increased to 369% in the presence of 5 mM ADP (Fig. 6). To examine changes in the kinetic parameters of *Sy*CS, the saturation curves of *Sy*CS for both substrates in the presence of 5 mM ADP were drawn (Fig. 3). In the presence of 5 mM ADP, the *K*_m_ value of *Sy*CS for oxaloacetate was 73 ± 11 μM, the *k*_cat_ value of *Sy*CS for oxaloacetate was 3.35 ± 0.54 s^−1^, and the *k*_cat_/*K*_m_ value of *Sy*CS for oxaloacetate increased to 45.65 ± 0.84 s^−1^mM^−1^ (Table 1). In the presence of 5 mM ADP, the *K*_m_ value of *Sy*CS for acetyl-CoA was 153 ± 24 μM, the *k*_cat_ value of *Sy*CS for acetyl-CoA was 2.99 ± 0.12 s^−1^, and the *k*_cat_/*K*_m_ value of *Sy*CS for acetyl-CoA increased to 19.90 ± 2.82 s^−1^mM^−1^ (Table 1). Compounds related to the TCA cycle and amino acid metabolism [pyruvate (PYR), succinate (SUC), fumarate (FUM), l-malate (MAL), and l-aspartate (ASP)] and nucleotides (ATP, AMP, NADH, NAD^+^, NADPH, and NADP^+^) did not significantly alter *Sy*CS activity (Fig. 6).

## Discussion

We purified and biochemically characterised CS from the cyanobacterium *Synechocystis* sp. PCC 6803 in this study. The *k*_cat_ value of *Sy*CS was lower than those of reported CSs from heterotrophic bacteria (8–263 s^−1^)^23–26^ (Table 1). The *K*_m_ value of *Sy*CS for acetyl-CoA was similar to that of CS from *Arthrobacter* strain DS2-3R (200 μM)^26^ but higher than those of reported CSs from heterotrophic bacteria (14–200 μM)^23–30^ (Table 1). Similarly, the *K*_m_ value of *Sy*CS for oxaloacetate was higher than those of CSs from heterotrophic bacteria (4–20 μM)^23–30^ (Table 1). These observations indicate that *Sy*CS is an inefficient enzyme, similar to malate dehydrogenase from *Synechocystis* 6803 (encoded by *citH*, sll0891), which is involved in the oxidative TCA cycle (Fig. 1)^31^. In *Synechocystis* 6803, carbon flux through the TCA cycle is low under heterotrophic, photoheterotrophic, photomixotrophic, and photoautotrophic conditions in contrast to other metabolic pathways such as the glycolysis and oxidative pentose phosphate pathways^22,32–36^. The low activity of *Sy*CS may be a factor in the observed low carbon flux through the oxidative TCA cycle in *Synechocystis* 6803. Additionally, CSs from *Thermosulfidibacter takaii* ABI70S6T^37^ and *Desulfurella acetivorans*^38^ catalyse the backward reaction, the cleavage of citrate. *Sy*CS did not catalyse the backward reaction. This result suggests that *Sy*CS is not involved in the reductive TCA cycle (Fig. 1).

**Figure 7:**
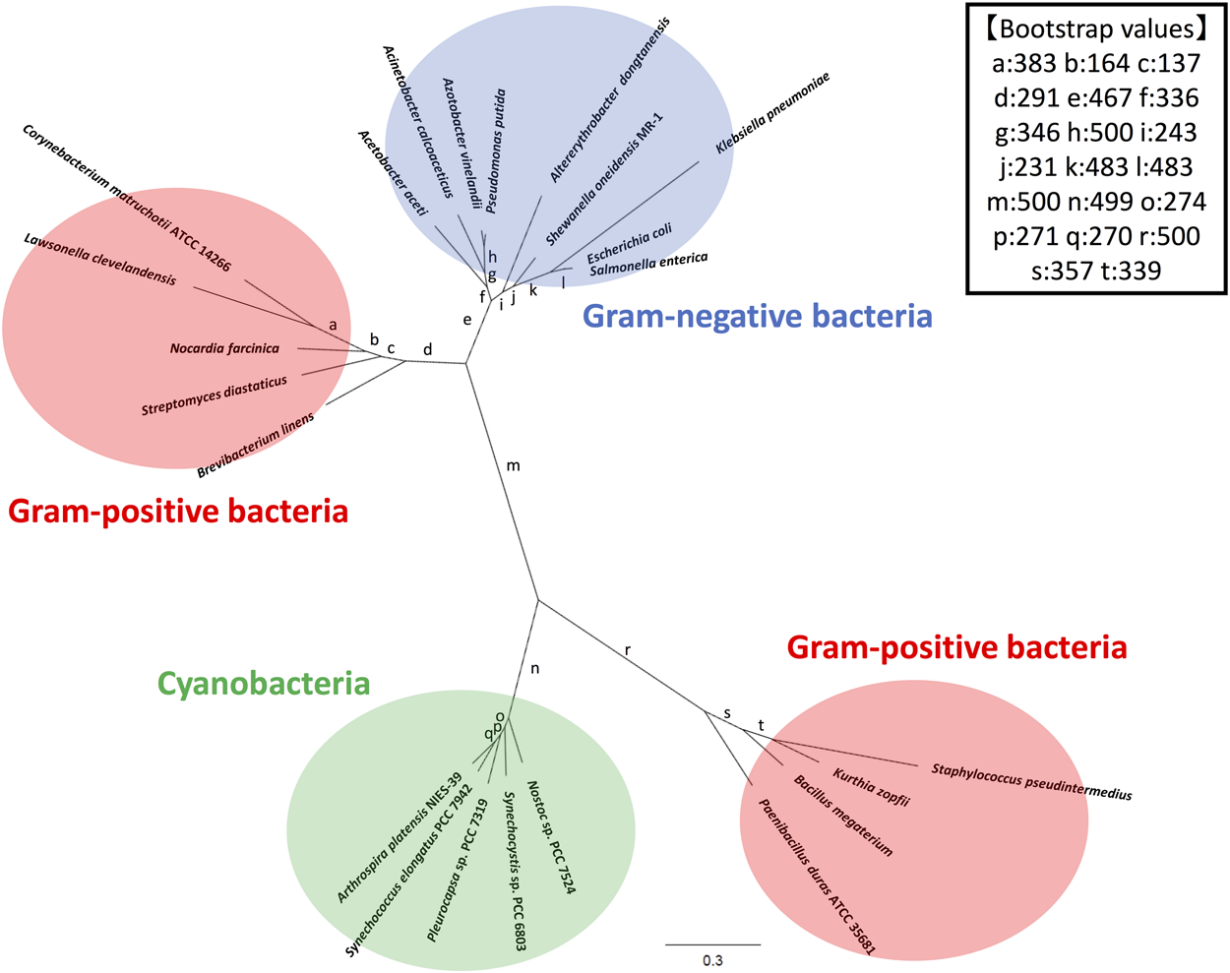
Phylogenetic analysis of bacterial CS. Sequences of 23 bacterial CS obtained from GenBank were aligned using CLC Sequence Viewer ver. 8.0. A maximum-likelihood tree based on 357 preserved amino acid residues was designed using PhyML online (http://www.atgc-montpellier.fr/phyml/). Bootstrap values were calculated by 500 replications.

Monovalent and divalent salts and cellular metabolites altered *Sy*CS activity (Figs 4–6). Monovalent salts activate numerous bacterial CSs^2^, while divalent salts inhibit many bacterial CSs^39,40^. In contrast to other bacterial CSs, *Sy*CS was markedly activated by the divalent salts MgCl_2_ and CaCl_2_ rather than monovalent salts (Fig. 4). Among divalent salts, MnCl_2_ inhibited *Sy*CS (Fig. 4). In CS from *E. coli*, a higher pH leads to greater activation by 0.1 M KCl^39^. In *Sy*CS, a higher pH led to greater activation by 0.1 M KCl and NaCl (Fig. 5a,b). In contrast, a lower pH led to greater activation by 0.1 M MgCl_2_ and CaCl_2_ (Fig. 5c,d). In monovalent and divalent salts, MgCl_2_ was the strongest activator of *Sy*CS (Figs 4 and 5). In the stroma in spinach chloroplasts, free Mg^2+^ concentration is affected by light-dark transition^41^. It is expected that free Mg^2+^ concentration in *Synechocystis* 6803 is similarly affected by light-dark transition^42^. Therefore, *Sy*CS activity may also be affected by light-dark transition. NADH is a feedback inhibitor of CSs in most Gram-negative bacteria^1^. However, *Sy*CS was not inhibited by NADH (Fig. 6). Phylogenetic analysis revealed that *Sy*CS belongs to a cyanobacterial clade being different from clades of Gram-positive bacteria and Gram-negative bacteria (Fig. 7). Unlike heterotrophic bacteria, cyanobacteria do not possess 2-oxoglutarate dehydrogenase in the TCA cycle, and *Synechocystis* 6803 is rerouted to produce succinate by two alternate pathways (Fig. 1)^43,44^. Isocitrate dehydrogenase from *Synechocystis* 6803 (encoded by *icd*, slr1289) (Fig. 1) is specific to NADP^+^ and generates NADPH but not NADH^45^. Additionally, malate dehydrogenase from *Synechocystis* 6803 (encoded by *citH*, sll0891) (Fig. 1) specifically catalyses the reductive reaction generating NAD^+^ but not NADH^31^. These previous studies explain why NADH does not function as a feedback inhibitor of *Sy*CS. CIT and 2OG are feedback inhibitors of *Sy*CS and several bacterial CSs^1,2,46^ (Fig. 6). We demonstrated that PEP is a unique inhibitor of *Sy*CS (Fig. 6). In the presence of PEP, the *K*_m_ values of *Sy*CS for both substrates were unchanged and the *k*_cat_ values of *Sy*CS for both substrates were decreased (Table 1). These results indicate that PEP is a non-competitive inhibitor of *Sy*CS. Phosphoenolpyruvate carboxylase from *Synechocystis* 6803 (encoded by *pps*, sll0920) catalyses the carboxylation of PEP to generate oxaloacetate, a substrate of *Sy*CS (encoded by *gltA*, sll0401) and malate dehydrogenase (encoded by *citH*, sll0891) (Fig. 1)^11^. The reaction catalysed by phosphoenolpyruvate carboxylase (encoded by *pps*, sll0920) (Fig. 1) is the rate-limiting step in the production of succinate via the reductive TCA cycle in *Synechocystis* 6803^9,10^. PEP has been suggested to regulate both the oxidative and reductive TCA cycle in *Synechocystis* 6803. The intracellular concentrations of CIT and PEP are much higher than that of 2OG (CIT: 7.3 times, PEP: 8.5 times)^13^. In inhibitors of metabolites, CIT and PEP may have large inhibitory effects on *Sy*CS under *in vivo* conditions. While ATP did not alter *Sy*CS activity (Fig. 6), it acts as a feedback inhibitor for other CSs because of its structural similarity with acetyl-CoA^1,2^. In terms of activators, AMP upregulates the activity of CSs from *Azotobacter vinelandii*^47^ and *Streptomyces diastaticus* No. 7 strain M1033^24^. However, *Sy*CS was activated by ADP rather than AMP (Fig. 6). ADP enhanced the catalytic efficiency (*k*_cat_/*K*_m_) of *Sy*CS (Table 1). Generally, ADP inhibits CS for the same reason as ATP^1,2^. Our study indicates that ADP is a unique activator of *Sy*CS. *Synechocystis* 6803 has more ADP than ATP in the cells^13^ in contrast to *E. coli*^48^ whose CS is inhibited by ATP and hardly affected by ADP^49^. This may be a reason that *Sy*CS is affected by not ATP but ADP.

Taken together, we found that the characteristics of *Sy*CS significantly differ from those of other bacterial CSs.

## Methods

### Construction of the recombinant *Sy*CS expression vector

The region containing *gltA* (sll0401, encoding *Sy*CS) in the *Synechocystis* 6803 genome was commercially synthesized by Eurofin Genomics Japan (Tokyo, Japan) and cloned into the BamHI-XhoI site of the pGEX5X-1 vector (GE Healthcare, Little Chalfont, UK).

### Affinity purification of recombinant proteins

The expression vector was transformed into *E. coli* DH5α cells (TakaraBio, Shiga, Japan). A 2-L culture of transformed DH5α cells in LB media was prepared by shaking at 125 rpm at 30 °C overnight. Expression of the recombinant proteins was induced by 0.01 mM isopropyl β-d-1-thiogalactopyranoside (Wako Chemicals, Osaka, Japan). Affinity purification of the recombinant proteins was performed as described previously^12^. The DH5α cells were lysed by sonication (VC-750, EYELA, Tokyo, Japan) for 200 s at 20% intensity. The supernatant was collected after centrifugation (5,800 × *g* for 2 min at 4 °C) and 560 μL of Glutathione-Sepharose 4B resin (GE Healthcare) was added to the supernatant. The mixture was incubated on ice for 30 min with constant shaking. After 30 min, 1 mM ATP and 1 mM MgSO_4_·7H_2_O were added to the mixture and shaken for 30 min at 37 °C. After centrifugation (5,800 × *g* for 2 min at 4 °C) to remove the supernatant, the resin was re-suspended in 700 μL of PBS-T (1.37 M NaCl, 27 mM KCl, 81 mM Na_2_HPO_4_·12H_2_O, 14.7 mM KH_2_PO_4_, and 0.05% Tween-20) with 1 mM ATP/1 mM MgSO_4_·7H_2_O. The resin was washed 10 times using PBS-T, and the recombinant proteins were eluted with 700 μL of GST elution buffer (50 mM Tris-HCl, pH 8.0, and 10 mM reduced glutathione) four times. The eluted protein fractions were concentrated using a VivaSpin 500 MWCO 50000 device (Sartorius, Göttingen, Germany). The protein concentration was measured using a Pierce BCA Protein Assay Kit (Rockford, IL, USA). To evaluate protein purity, SDS-PAGE followed by staining with InstantBlue (Expedion Protein Solutions, San Diego, CA, USA) was performed.

### Enzyme assays

The enzyme activity of *Sy*CS for the forward reaction, generating citrate and CoA from acetyl-CoA and oxaloacetate, was assessed as described previously^50^. Enzyme activity was calculated by measuring the change in *A*_412_ using a Hitachi U-3310 spectrophotometer (Tokyo, Japan). The purified *Sy*CS proteins were added to 1 mL assay solution [50 mM Tris-HCl containing various concentrations of oxaloacetate, various concentrations of acetyl-CoA, and 0.2 mM DTNB]. The *K*_m_ and *V*_max_ values of *Sy*CS were calculated using a Lineweaver-Burk double reciprocal plot. The *k*_cat_ values of *Sy*CS were determined from the *V*_max_ values of *Sy*CS. The enzyme activity of *Sy*CS for the backward reaction, generating acetyl-CoA and oxaloacetate from citrate and CoA, was assessed as described previously^38^ by measuring the change in *A*_365_ using a Hitachi U-3310 spectrophotometer. The purified *Sy*CS proteins were added to 1 mL assay solution [50 mM Tris-HCl containing 1 mM CoA, 1 mM citrate, 5 mM MgCl_2_, 0.5 mM NADH, and 20 U malate dehydrogenase (Oriental Yeast, Tokyo, Japan)].
